# The Fornix May Play a Key Role in Korsakoff’s Amnesia Secondary to Subcallosal Artery Infarction

**DOI:** 10.3390/brainsci12010021

**Published:** 2021-12-24

**Authors:** Masataka Hayashi, Ayataka Fujimoto, Hideo Enoki, Keiko Niimi, Chikanori Inenaga, Keishiro Sato, Kazunari Homma, Tomoya Arakawa, Tohru Okanishi

**Affiliations:** 1 Department of Neurosurgery, Seirei Hamamatsu General Hospital, Hamamatsu 430-8558, Japan; mhayashi@sis.seirei.or.jp (M.H.); inenaga@sis.seirei.or.jp (C.I.); t.arakawa@sis.seirei.or.jp (T.A.); 2 Comprehensive Epilepsy Center, Seirei Hamamatsu General Hospital, Hamamatsu 430-8558, Japan; enokih.neuropediatr@gmail.com (H.E.); k-sato@sis.seirei.or.jp (K.S.); t.okanishi@tottori-u.ac.jp (T.O.); 3 Department of Rehabilitation, Seirei Hamamatsu General Hospital, Hamamatsu 430-8558, Japan; k-niimi@sis.seirei.or.jp; 4 Department of Neurology, Seirei Hamamatsu General Hospital, Hamamatsu 430-8558, Japan; honma-k@sis.seirei.or.jp

**Keywords:** subcallosal artery infarction, Korsakoff’s amnesia, interventional radiology therapy, aneurysm, confabulation, memory disturbance

## Abstract

Background: Subcallosal artery infarction injures the fornix and anterior corpus callosum and sometimes causes Korsakoff’s amnesia. We hypothesized that Korsakoff’s amnesia might be caused by fornix dysfunction rather than anterior corpus callosum dysfunction in subcallosal artery infarction. Methods: A systematic review approach was applied to search PubMed and Google Scholar for articles to compare patients who had both bilateral fornix and corpus callosum infarction due to subcallosal artery territory ischemia (vascular event group; V group) with patients who had undergone anterior corpus callosotomy (callosotomy group; C group). Results: The V group comprised 10 patients (mean age, 63 years; median, 69 years; standard deviation (SD), 14.5 years; 5 males, 5 females). The C group comprised 6 patients (mean age, 23.7 years; median, 20 years; SD, 7.3 years; 3 males, 3 females). Six of 10 patients (60%) with subcallosal artery infarction exhibited Korsakoff’s amnesia. One patient showed neither confabulation nor amnesia. Conversely, no amnesia episodes were seen in any patients from the C group (*p* = 0.034). Conclusion: Fornix injury, rather than anterior corpus callosum injury, might be the major cause of Korsakoff’s amnesia in patients with subcallosal artery infarction.

## 1. Introduction

Wernicke’s encephalopathy due to thiamine deficiency is well known to lead to Korsakoff’s amnesia, in what is known as Wernicke-Korsakoff syndrome. However, in addition to Wernicke’s encephalopathy, sleep apnea syndrome [1] and heart failure [2] are also known to cause Korsakoff’s amnesia [3]. Other causes such as vascular events could therefore be expected to cause Korsakoff’s amnesia.

The subcallosal artery originates from the posterior or posterosuperior part of the anterior communicating artery (AcomA), with a length of 2.5 ± 1 mm, and a diameter of 0.8 ± 0.6 mm, supplying the paraterminal and paraolfactory gyri, rostrum and genu of the corpus callosum, lamina terminalis, and anterior commissure [4,5]. According to Chenin et al. [4], vascularization of the fornix by subcallosal arteries was seen in 5 of their 21 cases (24%). They also stated that subcallosal arteries vascularizing the fornix were significantly longer and had significantly larger diameters, and some subcallosal arteries led to peri-callosal arteries. Another study showed variants of subcallosal arteries with a C-shape (56%), straight course (17%) or S-shape (27%). Subcallosal arteries with a straight course displayed no perfusion of the fornix [6]. However, case reports have described patients with basal forebrain infarction secondary to subcallosal artery infarction resulting in amnesia [7,8,9,10]. Since the subcallosal artery has variants, the parts injured in subcallosal artery infarction would depend on the specific territories exclusively supplied by the subcallosal artery. The perfusion territories of the subcallosal artery are the genu and rostrum of the corpus callosum, anterior commissure, paraterminal gyrus (subcallosal gyrus), and cingulum with or without the anterior part of the fornix. Subcallosal artery injury can therefore exhibit variable symptoms, including amnesia, disturbance of consciousness, personality changes, dysphonia, and Korsakoff’s syndrome [11]. Based on these facts, one paper pointed out that “basal forebrain amnesia” was an erroneous term [12]. By this reasoning, the term “amnestic syndrome of the subcallosal artery” [13] could also be considered erroneous. As dysfunction of the corpus callosum itself might cause amnesia [14,15], we cannot conclude that amnesia from subcallosal artery area infarction is simply due to injury to the anterior fornix. The amnesia might instead be due to mixed dysfunction of the anterior fornix and anterior corpus callosum. According to Meila et al., patients with subcallosal artery infarction showing Korsakoff’s amnesia showed both fornix and anterior corpus callosum infarctions [16]. Another case report showed a patient with Korsakoff’s amnesia who displayed complete disconnection of the corpus callosum genu on diffusion tensor imaging [17]. However, whether either the fornix or anterior corpus callosum is responsible for Korsakoff’s amnesia secondary to subcallosal artery stroke has not been clarified.

We hypothesized that Korsakoff’s amnesia following subcallosal artery infarction might be caused by fornix dysfunction rather than anterior corpus callosum dysfunction. The purpose of this work was to review the published literature along with a local case of subcallosal artery injury and Korsakoff’s amnesia, comparing patients with subcallosal artery injury to our cases that had undergone corpus callosotomy to determine the validity of our hypothesis.

## 2. Methods

### 2.1. Study Design and Ethics Approval

The ethics committee at Seirei Hamamatsu General Hospital approved the protocol for this study (approval no. 3608), which was performed in accordance with the principles of the Declaration of Helsinki. The participant from our hospital provided written informed consent prior to inclusion in the study. We did not perform any interventions in the course of this study. For other participants, a systematic review approach was performed to identify relevant articles in the literature using PubMed and Google Scholar.

### 2.2. Clinical Information

We systematically reviewed patients with subcallosal artery infarction. Since we had a case that we studied separately from the rest of the study subjects identified from the literature, we included that case in the present review. We also retrospectively reviewed our electronic medical records to identify patients who had undergone anterior corpus callosotomy.

### 2.3. Identification, Screening, and Inclusion of Patients

We searched for the key terms “subcallosal artery” and “infarction” in articles published up to 20 October 2021, using PubMed and Google Scholar. We identified both cases of bilateral fornix infarction and cases of anterior corpus callosum infarction, including our case.

The exclusion criterion was subcallosal artery infarction due to ruptured aneurysm, because amnesia in such cases might be related to subarachnoid hemorrhage causing effects such as increased intracranial pressure and vasospasm-induced ischemic symptoms.

We had encountered a patient with subcallosal artery infarction after AcomA aneurysmal coil embolization. We compared patients who had both bilateral fornix and corpus callosum infarction due to subcallosal artery territory ischemia (lesion of the corpus callosum + fornix vascular event group; C + F group) with patients after corpus callosotomy (callosotomy group; C group) as a primary outcome.

In the C group, we retrospectively reviewed patients with epilepsy who: (1) had undergone anterior callosotomy; (2) showed Wechsler adult intelligence scale (WAIS)-IV ≥ 80 (normal intelligence) preoperatively; and (3) postoperative WAIS-IV or Wechsler intelligence scale for children (WISC)-IV evaluation a year after corpus colostomy from 2011 to March 2020. The reason that patients had undergone corpus callosotomy was medically intractable generalized epileptic seizure without focality judging from seizure semiology and neuroimages. We also included a patient with isolated fornix infarction (F group) [18].

### 2.4. Definition of Korsakoff’s Amnesia

No consensus has been reached on the definition of Korsakoff’s syndrome [19]. The key symptoms of Korsakoff’s amnesia are considered to be: (1) chronicity; (2) memory disturbance; and (3) confabulation. In this study, patients who newly exhibited this triad were regarded as having Korsakoff’s amnesia [20,21,22].

### 2.5. Statistical Analysis

Student’s *t*-test, the Mann-Whitney U-test, and Fisher’s exact probability test were used, as appropriate. Statistical significance was set at *p* < 0.05. All analyses were conducted using Sigma plot software (Systat Software, San Jose, CA, USA).

## 3. Results

### 3.1. Clinical Information

**Figure 1:**
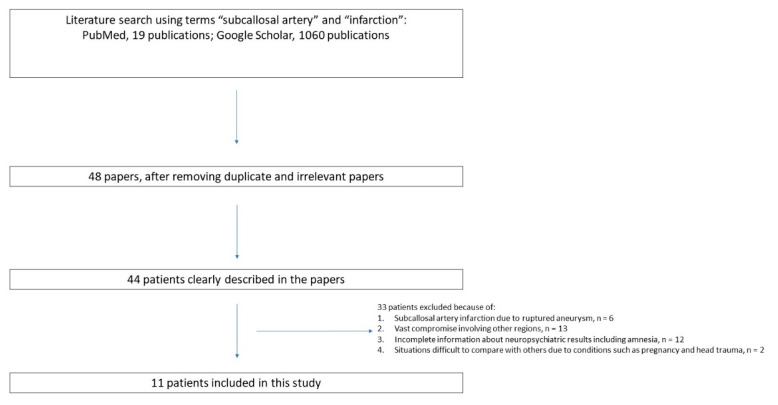
Systematic review flow diagram of this study.

A search for medical papers on PubMed and Google Scholar using the key words revealed 1079 papers (19 from PubMed, 1060 from Google Scholar) (Figure 1). The C + F group comprised 10 patients (mean age, 63 years; median, 69 years; standard deviation (SD), 14.5 years; 5 males, 5 females). The C group comprised 6 patients (mean age, 23.7 years; median, 20 years; SD, 7.3 years; 3 males, 3 females). These two groups showed a significant difference in age (*p* = 0.001), but not in sex (*p* = 1.000) (Table 1).

### 3.2. Korsakoff’s Amnesia

Six of the 10 patients (60%) [8,9,13,16,17,23,24,25,26] with subcallosal artery infarction exhibited Korsakoff’s amnesia. One patient (Case 8) showed neither confabulation nor amnesia. Conversely, no amnesia episodes were seen in the C group (*p* = 0.034). The fact that the one patient with isolated fornix infarction (F group) also exhibited confabulation appeared worth noting, given the hypothesis.

### 3.3. Our Case

The patient was a 69-year-old woman. She presented with dysphagia, and magnetic resonance angiography showed a 7-mm AcomA aneurysm. We planned to treat this aneurysm with interventional radiology (IVR) for coil implantation.

**Figure 2:**
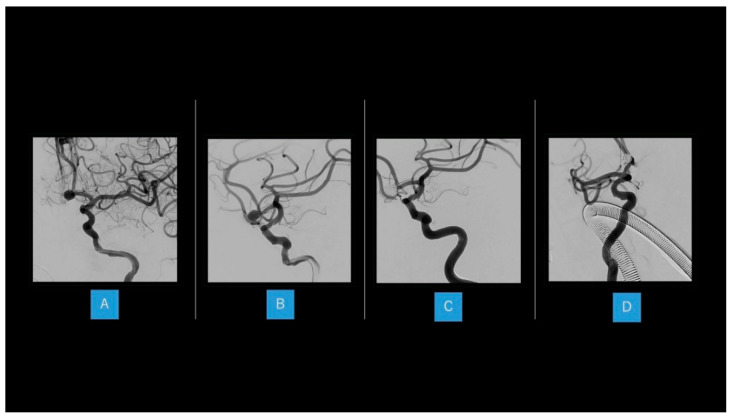
Interventional radiology therapy in our case (**A**,**B**) A 7 mm aneurysm is seen at the anterior communicating artery (AcomA). (**C**,**D**) Five coils are applied to the dome of the AcomA aneurysm under general anesthesia.

**Figure 3:**
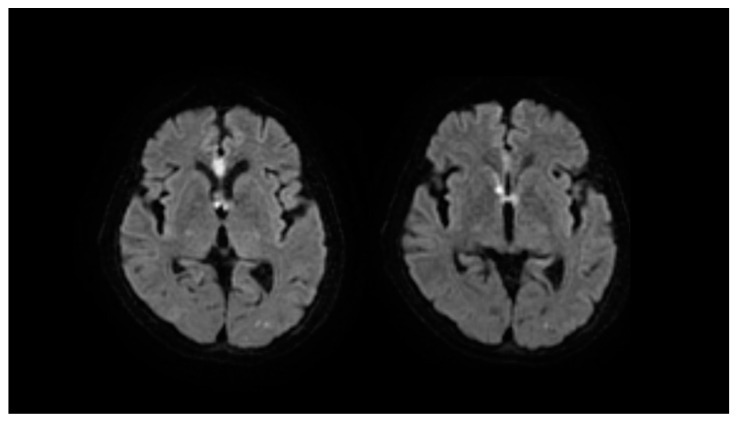
Diffusion-weighted imaging after coil embolization; in our case, high-intensity areas are seen at the anterior corpus callosum and bilateral fornix.

Dual antiplatelet therapy was started 2 weeks before the IVR therapy, five coils were applied to the dome of AcomA under general anesthesia. Volume embolization ratio was 29.8%, and postoperative 3-dimensional angiography showed complete occlusion (Figure 2). On postoperative day 1, the patient appeared slightly confused and displayed hemiparesis of the right upper limb. Diffusion-weighted imaging revealed new infarction of the anterior corpus callosum and bilateral fornix (Figure 3). Rehabilitation assessment showed cognitive dysfunction, particularly in the form of short-term memory disturbance. Memory symptoms continued until discharge, and remained at 1.5 years postoperatively.

Follow-up at 1.5 years postoperatively showed intact orientation of name, date of birth, age and place. However, the patient showed confusion regarding current date and which family members she was living with (Figure 3). The patient thought that an aunt who had passed away 10 years earlier was still alive and living with her. This phenomenon was considered to represent confabulation. Verbal memory as measured by Wechsler memory scale-revised was 75 (normal range 85–115), visual memory was 68 (normal range 85–115), and general memory was 70 (normal range 85–115).

No episodes of sleep apnea syndrome, or cardiac abnormality were seen in the patient. The patient also had no history of episodes of malnutrition or alcoholism that would suggest vitamin deficiency, although we did not measure thiamine levels at the time.

## 4. Discussion

The results of this study suggest that patients with Korsakoff’s amnesia who had bilateral fornix and anterior corpus callosum infarction due to subcallosal artery ischemia experienced Korsakoff’s amnesia mainly due to fornix injury, not injury to the anterior corpus callosum. Korsakoff’s amnesia is also seen with injury to the thalami and mammillary bodies [19,21,27,28,29]. The involvement of the fornix, mammillary bodies and thalamus in the memory network is explained by their status as components of the Papez circuit [11]. However, a patient who showed bilateral hippocampus disorder involving the Papez circuit after bilateral medial temporal lobectomy was not reported as having Korsakoff’s syndrome [30]. Since many reports of Korsakoff’s amnesia preceded by Wernicke encephalopathy have revealed atrophy of the fornix, thalami, and mammillary bodies, [19,31,32], the main structures involved in Korsakoff’s amnesia could be the fornix, thalami, and mammillary bodies.

One of the most prominent symptoms in Korsakoff’s syndrome is considered to be confabulation, which is related to limbic system disorder [33,34]. Patients with Korsakoff’s syndrome acted as if in a dream [35]. Confabulation is also related to dreams and perifornical regions could be related to sleep [36]. A sensation of being in a dream-like state while awake appears to be created by fornix injury in patients with subcallosal artery stroke, resulting in the exhibition of Korsakoff’s syndrome [37].

One patient (Case 8 in the C + F group) did not exhibit confabulation even though magnetic resonance imaging (MRI) revealed hyperintensity of the anterior fornix. Saito et al., who reported that case [25], differentiated the anterior fornix from the fornix and speculated that compromise of the anterior fornix might not show typical amnesia manifestation. MRI in Case 8 showed a larger injury to the anterior corpus callosum than other cases in the C + F group. As subcallosal arteries with a straight course reportedly display less perfusion of the fornix [6], patients with compromise of the subcallosal artery might not usually exhibit amnesia differently depending on vascularization. Since the patient in Case 8 had suffered from amnesia for 7 weeks before recovering from the symptoms, this patient might be considered to have followed an atypical clinical course for the C + F group.

Since patients with subcallosal artery stroke are rare, the size of the patient cohort in this study was insufficient to reach any definitive conclusions. In terms of future work, a study involving multiple facilities and considering amnesia caused by factors such as vascular injuries, sleep apnea, and cardiac dysfunctions is warranted.

## 5. Conclusions

Fornix injury, but not anterior corpus callosum injury, might be the major contributor to Korsakoff’s amnesia in patients with subcallosal artery infarction.

## Figures and Tables

**Table 1:** Collected cases.

					Injured Area		Clinical Manifestation		
	Case		Age *	Sex	Corpus Callosum	Fornix	Confabulation	Amnesia	
C + F group	1	Corpus callosum + Fornix lesion case 1	43	Male	+	+	+	+	Michel et al., 2020
	2	Corpus callosum + Fornix lesion case 2	79	Male	+	+	−	+	Pardina-Vilella et al., 2018
	3	Corpus callosum + Fornix lesion case 3	74	Female	+	+	+	+	Turine, G et al., 2016
	4	Corpus callosum + Fornix lesion case 4	71	Male	+	+	+	+	Meila et at., 2015
	5	Corpus callosum + Fornix lesion case 5	68	Male	+	+	+	+	Renou et al., 2008
	6	Corpus callosum + Fornix lesion case 6	33	Female	+	+	−	+	Hattingen et al., 2007
	7	Corpus callosum + Fornix lesion case 7	61	Male	+	+	+	+	Moussouttas et al., 2005
	8	Corpus callosum + Fornix lesion case 8	71	Female	+	+	−	−	Saito et al., 2006
	9	Corpus callosum + Fornix lesion case 9	60	Female	+	+	−	+	Park et al., 2000
	10	Corpus callosum + Fornix lesion case 10	70	Female	+	+	+	+	Our case
C group	1	Anterior callosotomy case 1	24	Female	+	−	−	−	
	2	Anterior callosotomy case 2	16	Male	+	−	−	−	
	3	Anterior callosotomy case 3	51	Male	+	−	−	−	
	4	Anterior callosotomy case 4	8	Female	+	−	−	−	
	5	Anterior callosotomy case 5	5	Male	+	−	−	−	
	6	Anterior callosotomy case 6	38	Female	+	−	−	−	
F group	1	Isolated fornix infarction case 1	61	Male	−	+	+	+	Salvalaggio et al., 2018

Note: Age difference exists < 0.005 *.

## Data Availability

Considering our participants’ privacy, no data are publicly available for this study.
